# Determinants of birthplace among middle-to lower-class women in Indonesia: A study using the Indonesian Demographic and Health Survey

**DOI:** 10.1371/journal.pone.0259417

**Published:** 2021-10-29

**Authors:** Angga Kresna Pranata, Andri Setiya Wahyudi, Lukman Handoyo, Ferry Efendi

**Affiliations:** 1 Faculty of Nursing, Universitas Airlangga, Surabaya, Indonesia; 2 Department of Nursing, Sekolah Tinggi Ilmu Kesehatan Widya Dharma Husada Tangerang, Tangerang, Banten, Indonesia; University of Mississippi Medical Center, UNITED STATES

## Abstract

**Background:**

One of the factors contributing to a high maternal mortality rate is the utilization of non-healthcare facilities as a birthplace for women. This study analyzed determinants affecting birthplace in middle-to lower-class women in Indonesia.

**Methods:**

This study analyzed the 2017 Indonesian Demographic and Health Survey (IDHS) data. The total national sample size was 49,627 eligible women. Our sample included 11,104 women, aged 15–49, who had delivered babies and were of low-to-middle economic status. The type of survey dataset was individual record dataset. Data were analyzed with chi-square and multivariate logistic regression tests using Stata 16 software.

**Results:**

About 64.99% middle to lower class women in Indonesia delivered in healthcare facilities. Women aged 45–49 (OR = 2.103; 95% CI = 1.13–3.93), who graduated from higher schools (OR = 2.885; 95% CI = 1.76–4.73), whose husbands had higher education (OR = 2.826; 95% CI = 1.69–4.74) and were employed (OR = 2.523; 95% CI = 1.23–5.17), who considered access to healthcare facilities was not a problem (OR = 1.528; 95% CI = 1.28–1.82), who had a single child (OR = 2.349; 95% CI = 1.97–2.80), and who lived in urban areas (OR = 2.930; 95% CI = 2.40–3.57) were determinants that significantly correlated with women giving birth in healthcare facilities.

**Conclusion:**

This study provides insights for policymakers and healthcare centers in the community to strengthen access to healthcare services and devise health promotion strategies for pregnant mothers. Policy interventions designed for middle- to lower-class women should be implemented to support vulnerable groups.

## Introduction

The choice of birthplace is important for pregnant mothers, as it affects maternal and infant mortality [1, 2]. Women who choose to give birth in healthcare facilities have a lower risk of complications than those who choose non-healthcare facilities. In healthcare facilities, professionals and trained health workers can assist mothers with advanced medical equipment [3]. Mothers who deliver in non-healthcare facilities may be at risk of delayed management of complications, such as postpartum infection and bleeding, due to the lack of medical equipment and trained birth assistants [4].

The impact on maternal mortality of low utilization of health facilities for delivery is a concern of the United Nations’ Sustainable Development Goals (SDGs). According to the World Health Organization (WHO), the global maternal mortality rate (MMR) in 2017 reached 211 per 100,000 live births [5]. The SDGs aim to reduce the MMR to less than 70 per 100,000 live births by 2030 [6]. Hence, actions are required to reduce maternal deaths by 68% in less than ten years. Developing countries, including Indonesia, contribute significantly to the global MMR. The Indonesian Ministry of Health reports that the 2015 MMR was 305 per 100,000 live births [7]. This rate is higher than other Southeast Asian countries. The target in 2015 was 102 per 100,000 live births. Recently, based on the SDGs program, the Indonesian Ministry of Health has renewed the MMR target to 131 per 100,000 live births (a decrease of 43%) by 2030. The 2015 target required strong cooperation and hard work across sectors since Indonesia had successfully reduced the MMR by 22% within 25 years (from 1991–2015) [7].

The main causes of a high MMR are preventable [8], especially if timely measures are taken. A WHO study identified three common causes of maternal mortality worldwide: bleeding, hypertension, and sepsis [9]. Bleeding and sepsis can be prevented through appropriate birthplace selection, which, in this case, is a healthcare facility, not a home [10]. Birthplace selection in Indonesia depends on several factors, including a woman’s economic status. Richer women may deliver in hospitals or primary healthcare clinics, while those with low-to-middle economic status tend to deliver at home to save costs [11]. The 2017 Indonesian Demographic and Health Survey (IDHS) showed that the percentage of poorer women who delivered in healthcare facilities increased from 57.2% to 66.6% between 2012 and 2017 [12, 13]. This should have reduced the MMR in Indonesia. However, other factors contributing to the MMR need investigation. Studies have shown that maternal deaths may occur due to delayed clinical decisions (referrals taking longer), inadequate birth assistants’ monitoring of women’s conditions, and the absence of physicians during labor and delivery [14, 15]. The studies indicate that maternal deaths mostly occur in community healthcare centers. The IDHS supports this assumption, showing that more than 50% of mothers with low–middle economic status prefer to give birth in community healthcare centers compared to hospitals [13]. This means that human resource management and readiness in these facilities should be evaluated.

Delivery site among middle to lower class women affects the utilization of maternal services either in health facilities or non-health facilities, despite economic affordability and regardless of government health insurance. Studying lower- and middle-class women is informative. Firstly, they are more vulnerable than women of a higher economic class. Secondly, it is interesting to see data on whether the lower and middle economic class utilized health care facilities frequently as intended by the government. Thirdly, this study may give insights into whether people receive the requisite healthcare from pro-poor programs, particularly maternal and child health. Some determinants that may affect women’s delivery include demographic factors of the husband and wife, household, and geographical factors that need further investigation. These determinants need to be clarified since there are no studies demonstrating their relationship with women’s preferences. Therefore, this present study analyzed determinants that may be related to birthplace preferences among middle- to lower-class women in Indonesia.

## Materials and methods

### Design and data source

This study used secondary data from the 2017 IDHS. The study included women who had delivered babies in the five years preceding the survey and consented to be interviewed. The mothers were aged 15–49 and were of low–middle economic status. Lower- and middle-income status was based on the wealth quintile calculation from the lowest to the middle quintile. Details of the calculation and construction of the wealth index can be found in the literature [16]. The final number of respondents was 11,104 mothers out of 49,627 eligible women. The independent woman-related variables included age (divided into seven ranges), education level (no education, primary, secondary, and higher), and employment status. Husband-related factors included age (six age groups), education level (no education, primary, secondary, and higher), and employment status. Other factors included geographical factors, such as the perception of access to healthcare facilities and place of residence (percentage of respondents who disclosed serious problems in accessing health care), and household factors, such as the number of children. The dependent variable was the women’s birthplace, categorized into delivery at healthcare facilities (government hospital, clinic, health center, village midwife, or private healthcare providers) and non-healthcare facilities (delivery at respondent’s home or other home). All non-healthcare facilities deliveries were assisted by unskilled attendants.

### Research tools

This study used the survey questionnaires distributed in 2017, the most recent survey. The questionnaires centered around four main topics: households, reproductive women, married men, and male adolescents. Specifically, the questionnaires employed were surveying reproductive-aged Indonesian women between 15 and 49 years. These questionnaires have explored some topical issues, which have also become of international interest. IDHS employs a complex sampling design with rural/urban stratification before randomly sampling the household.

### Statistical analysis

The statistical analysis was carried out using Stata version 16. (StataCorp, College Station, TX, USA). This study employed a chi-square test to identify the correlation between the independent variables and delivery site. In the multivariate analysis, a binary logistic regression test was carried out to identify determinants of birthplace. The results of the multivariate analysis were presented as odds ratio with a 95% confidence interval.

### Ethical considerations

Permissions to use the IDHS 2017 data was granted from the DHS MEASURE website. The ethical approval was obtained from ICF International’s Institutional Review Boards (IRBs). The survey ensured international ethical standards of privacy, confidentiality, anonymity, as well as informed consent. Further information regarding the ethical standard conducted by DHS can be found here https://www.dhsprogram.com/methodology/Protecting-the-Privacy-of-DHS-Survey-Respondents.cfm.

## Results

### Characteristics of the respondents

As shown in Table 1, compared to other age groups, women who preferred giving birth at non-healthcare facilities, the majority were aged 45–49 (46.84%). Furthermore, women who never attended primary schools (67.47%) preferred to deliver in non-healthcare facilities. Geographically, those who preferred giving birth at non-healthcare facilities (43.12%) lived in rural areas and perceived access to healthcare facilities as the constraint (45.17%). Regarding households, the percentage of women with three children who preferred to deliver in non-healthcare facilities was higher (43.88%). Further information about the respondents’ characteristics is available in Table 1.

**Table 1:** Demographic characteristics and birthplace preferences (N = 11,104).

Variable	Birthplace preferences n (%)			
	Non-healthcare facilities		Healthcare facilities	
**Age of mothers** (X^2^ = 11.7, p = 0.0363)	n	%	n	%
15–19	119	37.78	196	62.22
20–24	689	34.28	1321	65.72
25–29	972	34.64	1834	65.36
30–34	968	34.49	1839	65.51
35–39	727	35.17	1340	64.83
40–44	323	35.53	586	64.47
45–49	89	46.84	101	53.16
**Age of husband/spouse** (X^2^ = 21.5, p = 0.029)				
Less than 19	22	38.60	35	61.40
20–29	1013	35.57	1835	64.43
30–39	1821	33.84	3560	66.16
40–49	863	35.49	1569	64.51
50–59	152	44.31	191	55.69
60+	16	37.21	27	62.79
**Mother’s education level** (X^2^ = 496.29, p<0.001)				
No education	168	67.47	81	32.53
Primary	1660	44.82	2044	55.18
Secondary	1796	29.20	4354	70.80
Higher	263	26.27	738	73.73
**Husband’s education level** (X^2^ = 319.11, p<0.001)				
No education	154	63.64	88	36.36
Primary	1672	42.32	2279	57.68
Secondary	1862	30.35	4273	69.65
Higher	199	25.64	577	74.36
**Mother’s employment status** (X^2^ = 14.4, p = 0.0258)				
No	1936	34.06	3748	65.94
Yes	1951	36.00	3469	64.00
**Husband’s employment status** (X^2^ = 10.2, p = 0.0301)				
No	45	44.55	56	55.45
Yes	3842	34.92	7161	65.08
**Perception of access to healthcare facilities** (X^2^ = 141.17, p<0.001)				
Big problem	757	45.17	919	54.83
Not a big problem	3130	33.20	6298	66.80
**Number of children** (X^2^ = 305.77, p<0.001)				
1	737	26.14	2082	73.86
2	1094	30.39	2506	69.61
≥ 3	2056	43.88	2629	56.12
**A place of residence** (X^2^ = 539.97, p<0.001)				
Rural	3125	43.12	4123	56.88
Urban	762	19.76	3094	80.24
**Total (n = 11,104 births)**	3887	35.01	7217	64.99

### Chi-square test

The chi-square tests conducted showed that the following variables were significantly related to women’s preferences for choosing birthplace: women’s education level (p<0.001), husbands’ education level (p<0.001), women’s employment status (p = 0.0258), husband’s employment status (p = 0.0301), perception of access to healthcare facilities (p<0.001), the number of children (p<0.001), and place of residence (p<0.001) (Table 1).

### Multivariate analysis

The multivariate analysis was performed using binary logistic regression, as depicted in Table 2. Adjusted binary logistic regression showed that women aged 45–49 were twice as likely to deliver in healthcare facilities than mothers aged 15–19 (OR = 2.103; 95% CI = 1.13–3.93). Women who graduated from higher school were 2.88 times more likely to give birth in healthcare facilities than those who never graduated from primary schools (OR = 2.885; 95% CI = 1.76–4.73). Women whose husbands attended higher education were 2.82 times more likely to deliver in healthcare facilities than women whose husbands had no educational background (OR = 2.826; 95% CI = 1.69–4.74). Women whose husbands were employed were 2.5 times more likely to prefer healthcare facilities for delivery than mothers with unemployed husbands (OR = 2.523; 95% CI = 1.23–5.17). Women who did not perceive access to healthcare facilities as a constraint were 1.5 times more likely to choose healthcare facilities for delivery than those who perceived access as a problem (OR = 1.528; 95% CI = 1.28–1.82). Women who only had one child were twice as likely to choose healthcare facilities for their delivery than those with three or more children (OR = 2.349; 95% CI = 1.97–2.80). Those in urban areas were 2.9 times more likely to prefer healthcare facilities than those in rural areas (OR = 2.930; 95% CI = 2.40–3.57).

**Table 2:** Binary logistic regression of determinants affecting birthplace preferences.

Variable	Delivery site Preferences							
	Adjusted				Unadjusted			
	P	OR	95% CI		P	OR	95% CI	
			lower	upper			lower	upper
**Age of mother**								
15–19	*Ref*.				Ref.			
20–24	0.933	1.015	0.72	1.43	0.069	1.279	.981	1.668
25–29	0.571	1.106	0.78	1.57	0.002*	1.534	1.166	2.019
30–34	0.066	1.431	0.98	2.10	0.000*	1.985	1.484	2.655
35–39	0.001*	1.973	1.33	2.94	0.000*	2.302	1.700	3.117
40–44	0.001*	2.052	1.33	3.17	0.000*	2.519	1.804	3.517
45–49	0.020*	2.103	1.13	3.93	0.010*	1.789	1.152	2.779
**Age of husband/spouse**								
Less than 19	*Ref*.				Ref.			
20–29	0.614	1.243	0.53	2.90	0.733	1.107	.618	1.982
30–39	0.371	1.489	0.62	3.56	0.219	1.446	.803	2.605
40–49	0.243	1.685	0.70	4.05	0.070	1.740	.956	3.168
50–59	0.493	1.399	0.54	3.65	0.242	1.467	.772	2.787
60+	0.353	1.769	0.53	5.90	0.076	2.246	.919	5.489
**Mother’s education level**								
No education	*Ref*.				Ref.			
Primary	0.001*	2.077	1.37	3.15	0.000*	1.788	1.331	2.402
Secondary	0.000*	3.659	2.38	5.62	0.000*	2.743	2.034	3.701
Higher	0.000*	2.885	1.76	4.73	0.000*	2.638	1.878	3.704
**Husband’s education level**								
No education	*Ref*.				Ref.			
Primary	0.014*	1.654	1.11	2.47	0.002*	1.579	1.176	2.121
Secondary	0.000*	2.281	1.52	3.43	0.000*	1.955	1.451	2.633
Higher	0.000*	2.826	1.69	4.74	0.000*	2.394	1.688	3.396
**Mothers’ employment status**								
No	*Ref*.				Ref			
Yes	0.363	0.939	0.82	1.08	0.697	.983	.903	1.071
**Husbands’ employment status**	* *							
No	*Ref*.				Ref			
Yes	0.012*	2.523	1.23	5.17	0.004*	1.864	1.218	2.851
**Perception of access to healthcare facilities**	* *							
Big problem	*Ref*.				Ref			
Not a big problem	0.000*	1.528	1.28	1.82	0.000*	1.310	1.170	1.467
**Number of children**								
≥ 3	*Ref*.				Ref			
2	0.000*	3.043	2.46	3.76	0.000*	1.310	1.170	1.467
1	0.000*	2.349	1.97	2.80	0.000*	1.310	1170	1467
**Place of residence**								
Rural	*Ref*.							
Urban	0.000*	2.930	2.40	3.57	0.000*	2.786	2.532	3.066

* There is a significant relationship with delivery site preference

For unadjusted binary logistic regression, almost all variables were significant except for age of husband and mother’s employment status.

## Discussion

Birthplace preferences among middle-to lower-class women in Indonesia were related to husband, household, and geographical factors in addition to factors related to the women themselves. Our findings show that women aged 45–49 were more likely to choose healthcare facilities for delivery than those aged 15–19. This may be due to their previous delivery experiences. Older women tend to have either personal delivery experiences or have seen or heard of others [17]. For instance, women who had perhaps had a home delivery, but the infants were stillborn due to unskilled birth attendants. Therefore, the mothers then chose a safer delivery site [18]. Older women anticipate unexpected incidents during birth and thus, prefer healthcare facilities as their birthplace [19, 20]. Therefore, age maturity, experience, and knowledge of deliveries are important to support the birth process in healthcare facilities.

The women’s and husbands’ education levels were related to the choice of birthplace. Women who attended, or whose husbands attended, higher education were more likely to choose healthcare facilities for delivery than those who never graduated from primary school. This finding is supported by previous studies that have found a correlation between level of education and choice of birthplace [21–23]. A study in Western Ethiopia revealed that women and husbands with a high school education were four times more likely to prefer healthcare facilities for delivery than those who were never enrolled in formal education [21]. Higher education can influence better health choices since it imparts comprehensive knowledge and critical thinking [24]. Further, women and husbands with higher education levels are more likely to be exposed to maternal education.

This present study also shows that working husbands were more likely to prefer delivery at healthcare facilities than unemployed ones. Working husbands have a financial status that enables them to provide better options for their family’s health, including their wives’ maternal health [25]. In contrast, when husbands are unemployed, their wives might not be able to access welfare and fulfil their daily needs [26]. Research shows that the higher the family income, the more the use of maternal health service facilities [27]. The husband’s occupation may correlate with family income, as noted by some studies [23]. Therefore, increasing access to economic opportunities in every household is needed to improve access to healthcare services.

Household factors, including the number of children born, are also related to birthplace preferences. This study shows that women with one child tend to choose healthcare facilities as the birthplace compared to women with three children or more. Previous research has also found similar relationships; primiparous women and those with one or two children were more likely to deliver in healthcare facilities than multiparous women and those with at least five children [28, 29]. This may be due to better delivery preparation that primiparous women have during pregnancy [30]. In addition, the cost of living of women with one child is not as high as it is for women with at least three children. As a result, delivery at home may be preferable for women who have many children [29]. Deciding on the number of children to have is a couple’s reproductive right. However, awareness of the cost of fulfilling a family’s daily needs should be considered. This situation may be solved by strengthening family planning in the community, which focuses on low and middle-income families.

Concerning geographical factors, this study found that women who did not perceive access as a constraint were significantly correlated with the choice of a healthcare facility birthplace. In addition, women who lived in urban areas would have more possibilities to deliver in healthcare facilities. A study by Kenea and Jisha in 2017 [31] reported that urban mothers dominated delivery in healthcare facilities. This study also aligns with 2019 findings from Indonesia [32]. The disparity of the quantity and quality of healthcare between urban and rural areas of Indonesia is an issue [14, 33–35]. Increasing access to healthcare is fundamental, especially for women living in rural areas of Indonesia.

## Conclusions

Birthplace preferences among middle-to lower-class women in Indonesia correlate with several factors, including women’s age, level of education, husbands’ employment status, perceived access to healthcare facilities, the number of children, and place of residence. Delivery preference for non-healthcare facilities should be of concern to professional health workers and policymakers. The government’s health insurance scheme made available to poor communities can maximize its effectiveness by improving access to healthcare facilities. Despite access, health workers also need to conduct more widespread health promotion to women who are not enrolled in formal education to better understand the need for a healthy birthplace.
